# A Nudge-Inspired AI-Driven Health Platform for Self-Management of Diabetes

**DOI:** 10.3390/s22124620

**Published:** 2022-06-19

**Authors:** Shane Joachim, Abdur Rahim Mohammad Forkan, Prem Prakash Jayaraman, Ahsan Morshed, Nilmini Wickramasinghe

**Affiliations:** 1 Department of Computing Technologies, School of Science, Computing and Engineering, Swinburne University of Technology, Melbourne 3122, Australia; fforkan@swin.edu.au (A.R.M.F.); pjayaraman@swin.edu.au (P.P.J.); 2 College of Information and Communications Technology, School of Engineering and Technology, Central Queensland University, Melbourne 3000, Australia; a.morshed@cqu.edu.au; 3 Department of Health Sciences and Biostatistics, School of Health Sciences, Swinburne University of Technology, Melbourne 3122, Australia; nilmini.work@gmail.com; 4 Epworth Healthcare, Richmond 3121, Australia

**Keywords:** diabetes, self-management, nudge theory, co-design, development, digital health platform, mHealth

## Abstract

Diabetes mellitus is a serious chronic disease that affects the blood sugar levels in individuals, with current predictions estimating that nearly 578 million people will be affected by diabetes by 2030. Patients with type II diabetes usually follow a self-management regime as directed by a clinician to help regulate their blood glucose levels. Today, various technology solutions exist to support self-management; however, these solutions tend to be independently built, with little to no research or clinical grounding, which has resulted in poor uptake. In this paper, we propose, develop, and implement a nudge-inspired artificial intelligence (AI)-driven health platform for self-management of diabetes. The proposed platform has been co-designed with patients and clinicians, using the adapted 4-cycle design science research methodology (A4C-DSRM) model. The platform includes (*a*) a cross-platform mobile application for patients that incorporates a macronutrient detection algorithm for meal recognition and nudge-inspired meal logger, and (*b*) a web-based application for the clinician to support the self-management regime of patients. Further, the platform incorporates behavioral intervention techniques stemming from nudge theory that aim to support and encourage a sustained change in patient lifestyle. Application of the platform has been demonstrated through an illustrative case study via two exemplars. Further, a technical evaluation is conducted to understand the performance of the MDA to meet the personalization requirements of patients with type II diabetes.

## 1. Introduction

Diabetes (diabetes mellitus) is a rapidly growing chronic disease that is known to affect individuals typically over the age of thirty, all around the world [1]. In 2017, 425 million people were affected by diabetes [1]. This number increased to 463 million in 2019 and is predicted to continue increasing, to reach 578 million by 2030 [2]. In Australia, as of 2017–18, 1 in 20 Australians (1.2 million individuals) was living with diabetes and this figure is expected to only grow [3]. The current growth trend estimates that up to 3 million Australians over the age of 25 will have a form of diabetes by 2025—with 85% of all diabetes being type II diabetes [4].

The rise in diabetes can be credited to a combination of factors, which include a sedentary lifestyle, poor diet, lack of regular exercise, and stress [5]. With continued poor management, the complications caused by diabetes can worsen and can lead to severe health consequences [5]. Diabetes can be an unpleasant chronic condition, which only worsens if left unchecked and may require further invasive, ongoing, and expensive medical attention [5].

Treating diabetes and its consequences, when there is a clear absence of any effective cure, means to maintain healthy appropriate blood sugar levels by concentrating on adhering to a *balanced diet*, exercising regularly, ensuring timely intake of medication, and undergoing regular checkups with a physician [5]. As diabetes is a chronic disease, there is no general cure and thus, self-management becomes a key aspect in living with diabetes. A good self-management regimen can guide patients to improve their diabetes and stay away from avoidable complications that can develop due to poor and uncontrolled diabetes [6]. In some cases, a well-structured and personalized regimen can even effectively help permanently reverse type II diabetes [6].

Self-management regimens for patients with diabetes generally involve monitoring blood glucose levels and blood pressure daily and keeping these within the target ranges; eating a healthy diet focusing on foods with a low glycemic index (GI); engaging in regular physical activity; reducing weight if it is above the recommended range; and quitting smoking [5]. For example, increased physical activity alone is known to contribute to 30–50% reduction in the development of type II diabetes [7]. Though self-management regimens can produce positive outcomes in managing diabetes, a survey conducted with over 100 patients with diabetes suggested that a technological-driven solution may be a driver for improved diabetes self-management [8].

While there is currently a large variety of diabetes self-management solutions available in the relevant app stores (e.g., Google’s Play Store and Apple’s app Store), they typically vary in core diabetes self-management feature sets and, more importantly, lack advanced features, such as a nutrition system that tracks diet as well as provides appropriate diet recommendations based on a clinician-set plan. Nutrition management is an important part in self-managing diabetes, yet many solutions only offer basic nutrition management functionality, such as logging [9]. This is potentially putting individuals at risk, as the diet outcomes of the developed solution are not curated or validated by a clinician and the solution is generalized for all individuals, disregarding the cultural or ethnic nuances, which are critical in supporting the diversity in the user population. 

This identified variability in the core features between each of the developed solutions is found to result in poor usability and effectiveness [10]. To improve usability issues in diabetes self-management solutions, studies have suggested that solutions should implement behavioral intervention principles [11]. Implementing behavioral interventions stemming from *nudge theory* can affect dietary and self-management behavior in a positive way [12]. While evidence supports the importance of implementing behavioral interventions in self-management systems for a sustained change in user behavior, current developed systems are yet to incorporate this in their solutions.

To address the identified key voids in the diabetes self-management solutions, this paper proposes, develops, and implements a nudge-theory-inspired personalized diabetes self-management platform for patients with type II diabetes. In this paper, we present the implementation of this platform, which has been co-designed with clinicians and patients with type II diabetes, ensuring that the features of the platform provide for personalized self-management and the platform is fit for purpose. The co-design process identified that a holistic diabetes self-management platform is required to ensure that we address the gaps found in the current literature, as well as the plethora of diabetes self-management solutions currently available. Since type II diabetes directly correlates with poor lifestyle and nutrition management [5], to develop a holistic solution, the proposed self-management solution needs to contain ways to manage a patient’s nutrition, fitness, blood sugar levels, and medication. Further, the proposed holistic solution needs to incorporate some form of communication pipeline with a clinician to ensure the solution is clinically sound. This study proposes a diabetes self-management platform that contains the following: (*a*) A cross-platform mobile application (diabetes self-management mobile app) that is personalized to the patient. The patient will be using the app to self-manage their type II diabetes by logging their blood glucose readings, fitness activities, and nutrition intake and any medication that they may be taking; (*b*) A clinician web portal that will allow the clinician to view how the patient is progressing with their self-management regimen and curate a personalized patient-specific diet recommendation list. The curated recommendations are then presented to the patient through the diabetes self-management mobile app. This is identified as a nudge to improve the patient’s diet intake, which improves self-management of the patient’s diabetes. Further, a macronutrient detection algorithm (MDA) has been implemented into the diabetes self-management platform to better understand what the patient is consuming based on meal images. MDA is able to analyze the food images provided by the patient at the time of logging and presents the macronutrient information of the food item to the patient. By focusing on a nudge-inspired nutrition management system, over time, we aim to have a sustained change in the patient’s behavior and overall improvement of self-management.

In summary, the paper makes the following contributions:

- Presentation of a holistic diabetes self-management platform that has been co-designed with patients and clinicians to ensure the platform is fit for purpose. Through the co-design process, it was identified that a self-management platform requires a cross-platform mobile app that is personalized to the patient’s cultural nuances and a clinician web app that allows for an improved patient–clinician communication pipeline.
- The development and implementation of a macronutrient detection algorithm (MDA) that uses AI-driven image analytics for nutrition management and behavior intervention through the incorporation of nudge theory. The platform aims to improve self-management among patients and enable them to undergo a sustained behavior change. An application of the platform has been demonstrated through an illustrative case study. Further, a technical evaluation is conducted to understand the performance of the MDA to meet the personalization requirements of patients with type II diabetes.

The rest of the paper is organized as follows. Section 2 reviews the related work, while Section 3 provides an overview of nudge theory, and Section 4 is about the methodology. Section 5 details the architecture of this platform, while Section 6 provides the details on the platform implementation and evaluation of identified deep learning models for platform personalization. Section 7 discusses the application of the personalized diabetes self-management platform in use through an illustrative case study approach. Section 8 provides a discussion on the findings, with the conclusion and future works.

## 2. Related Work

### 2.1. Current mHealth Applications

In Australia, it is estimated that 84% of all adults and 99% of 18–29-year-olds own and have access to a smartphone [13]. Accessibility of smartphones has played a vital role in developing mHealth mobile applications that promote self-management of diabetes [14]. In modern mobile devices, running operating systems such as Google’s Android OS and Apple’s iOS, developers can leverage the plethora of the onboard sensors to better understand the user context, and extract useful information [14].

Across the respective iOS and Android application stores, it is estimated that there are upwards of 300,000 active mHealth apps [15]. For many of the advanced smartphone users, mHealth applications can be seen to benefit by assisting them with self-management of their chronic disease [16]. However, due to the open nature of the app stores, the mHealth apps are developed and published by individuals and/or third-party teams [16]. This raises questions around how the mHealth apps manage patient health data and any legal liabilities of damages caused by the app [10].

Additionally, due to the lack of regulations and support from authoritative bodies, clinicians find it difficult to discuss mHealth options that patients can incorporate into their clinically approved diabetes self-management routines [16]. This has led many commercially available mHealth apps to provide diabetes self-management capabilities, without any medical or research rigor, which ultimately could place the patient at risk of an unexpected complication [9,17].

While type II diabetes correlates with poor lifestyle and nutrition management [5], it is found that many of the identified self-management mobile apps found in the market lack advanced nutrition management features. While there are many apps that partially cover the logging features blood glucose management, fitness tracking, and nutrition, none of them provides a solution that covers all the vital features to allow for clinically sound self-management [9]. Further the current suite of apps lacks various personalization features, such as a nutrition recommendation system that allows for diet recommendations from a clinician-managed list [9]. The apps that take the generalized approach and provide only the basic functionality create the possibility of adverse complications in the user as they may reach a state of hyper- or hypoglycemia due to poor lifestyle and nutrition management.

### 2.2. Nutrition Recommendation

Managing nutrition is a one aspect of diabetes self-management. However, poor dietary habits and an inactive lifestyle are the major factors behind the rapid growth of type II diabetes all around the world [1,2,18]. While studies suggest that socioeconomic factors such as low income and less education can contribute to an individual developing type II diabetes, a well-balanced lifestyle, which includes eating not only the right type of food but also the appropriate portion size, as well as partaking in some regular physical activity, can contribute to lowering the chances of developing type II diabetes [5,18,19].

Recommendation systems, fundamentally, can be defined as systems that find items that are relevant to the user based on previous decisions that the user has taken [20]. While previous decisions can be used for recommendations, modern recommendation systems also have the ability to predict the preferences of unrated items and continue to recommend new items to the user [21]. While there are many nutrition recommendation systems present, such as those mentioned in [22,23,24], they can be bundled under certain overarching categories.

The categories are as follows:

- ***Collaborative Filtering Recommender (CF) Systems:*** CF systems are known to be one of the most researched recommender systems [21]. This system uses a nearest neighbors approach, where it identifies other users who have a similar taste in the recommendation items as the user and recommends items that the neighbors like [25]. For this system to work, the user needs to have rated some of the existing items so the CF system can learn what the user likes and does not like.
- ***Content-based Recommender (CB) Systems:*** CB systems analyze the profile metadata of a user (likes, dislikes, etc.) to create a criterion, which is then used to find items that fulfill the criterion [20,21]. Usually, this method involves a text-mining algorithm that allows the system to identify key terms to match with the criterion [20].
- ***Knowledge-based Recommender (KB) Systems:*** KB systems use knowledge about the user and items to pursue recommendations based on reasoning related to whether the items meet the requirements of the user [26]. An example in relation to nutrition would be already having information such as what dishes the user likes/dislikes, what ingredients the user likes or dislikes, known allergies, etc.
- ***Deep Learning Recommender (DL) Systems:*** DL systems can capture nonlinear and nontrivial relationship between the user and the item, which may not be as quickly identifiable through a CB, CF, or KB system [27,28]. DL systems are the newer recommender system in this list, as a survey suggests exponential growth in research publications around DL recommender systems [27].
- ***Hybrid Recommender (HR) Systems:*** HR systems are based on the combination of the above-mentioned techniques. Studies have found HR systems to be the preferred approach as these systems could allow one system to cover the disadvantages of the other system [18,29]. For example, a well-known problem with the CF system is a “cold-start” problem [30]. This problem highlights the lack of information about the user unless the user starts using the system so that over time the system improves its recommendations. A way to overcome the cold-start issue is by pairing the CF system with the KB or CB system. Given the nutrition space, one can see that now there is some foundational information inherited from the user (through CF/KB system strategies) even before the user is required to use the system to log and rate meals. This allows for improved recommendations from the very beginning.

A survey conducted by Abhari et al., in 2019, looked specifically at nutrition recommendation systems, and using the PRISMA framework, they were able to identify 25 articles that implement various types of NR systems [29]. The survey identified that hybrid and knowledge-based recommender systems are the popular recommendation system types, with the collaborative recommendation system being the least popular type [29]. This study also highlights that the K-Means clustering algorithm, a deep learning recommender system, is not as widely used as the rule- or ontology-based algorithms [29]. Mobile applications were seen to be the platform with the highest percentage of nutrition recommendation systems, at 28%, followed by web applications, at 20% [29].

Research has highlighted that having a balanced diet is a critical factor when it comes to successful diabetes management [5]. Hence, it is crucial to have a strong recommendation system in place that can lead to improved self-management. Currently, while there are many stand-alone recommendation systems available, they are yet to be adapted to the health context (e.g., diabetes self-management). If they were to be adapted to a health context, a study highlights the importance of using an iterative design cycle that will allow the developers to better understand the domain and come up with an accurate evaluation process that will consider data security, privacy, ethical implications, etc., and understand the inception—which refers to methods used to conceive the recommender system in a health context [20]. For example, a diabetes self-management nutrition recommendation system needs to consider things such as blood sugar level, fitness history, and medication before considering the “normal” factors for a nutrition recommendation system, which would be calories, carbs, fat content, etc.

Another study covering recommender systems in the healthy food domain highlights that, even though there are some papers that theorize promoting a healthy lifestyle through food recommendation systems to help tackle health problems and suggest a change in eating behavior, these solutions are yet to be developed [21].

### 2.3. Behavioral Intervention

While the above identifies some of the functionality of what is expected in a diabetes self-management mHealth app, research suggests that to have an ongoing sustained change, the mHealth app needs to incorporate behavioral intervention techniques [31]. While changes influenced by these interventions are not always drastic, they have consistently produced positive outcomes in studies [8,32]. Table 1 contains a collection of lifestyle-related studies with *nudging* interventions in place with their respective outcomes.

Patients with diabetes have been known to feel unmotivated when it comes to the self-management of their diabetes, and this lack of motivation itself is known to be one of the main reasons behind an individual’s diabetes worsening over time [38]. When behavioral intervention methods, such as nudge theory, are used for patients with diabetes, the patients tend to demonstrate improved glycemic control over the long term, compared to patients that were not exposed to any behavioral intervention techniques [31,39,40,41]. An mHealth diabetes intervention solution that implements such a behavioral intervention method can help in decreasing the frequency of visits to the physician’s office and general practitioner and improve medical adherence [33].

### 2.4. Summary of Gaps

This section is a summary of the gaps identified in existing diabetic self-management solutions. A study was conducted in which we reviewed some of the leading iOS and Android diabetic self-management applications [9]. As a part of this study, we searched the respective app stores using keywords such as “diabetes” and “self-management”, filtered based on total downloads and ratings, and identified 10 applications. These applications were then screened to view the feature set they provided, as well as to find out if the applications were backed by any research.

Table 2 contains information related to the identified applications, which are:

Research, Published: Does the application have any publications associated with it?

Research, Theory: Does the application have any theory associated with it (e.g., grounded theory)?

Feature set: Does the application have said feature?

The three symbols in Table 2 indicate the following: tick (✓): the described item exists or is complete; cross (✕): the described item does not exist or is incomplete; and strike (**–**): the feature is partially present or uses an external application to cater for this.

While type II diabetes is connected with poor lifestyle and nutrition management, most if not all of the current diabetes self-management applications cater for the above-identified areas completely. However, there are clear gaps in the areas of clinical support, nutrition, fitness logger, and behavioral intervention (BI). Furthermore, the identified applications lack research grounding and rigor. It is possible that not using appropriate theories and evaluation strategies could have added to the poor fit and tarnished the final developed solution. Due to this, some of the diabetes self-management applications could be deemed not fit for purpose and may fail to help patients better self-manage their diabetes. It can also be observed that none of the applications has a nutrition recommender feature. Improved nutrition management allows for improved glycemic control, which is crucial when ensuring the patient does not reach a state of hyper- or hypoglycemia [1,2,18]. Behavioral Intervention features are also poorly covered by these applications. With many research studies providing evidence supporting the benefits of implementing behavioral interventions as a part of a diabetes mHealth solution, the majority of the applications developed and available in the relevant app store [9] fail to address implementing behavioral interventions techniques that stem from tried-and-proven methods, such as nudge theory [44].

In contrast, this paper proposes a co-designed holistic diabetes self-management platform that will contain *fundamental features* that span blood glucose levels, nutrition, physical activity, medication, clinical services, and personalized features (e.g., nutrition recommendation, fitness recommendation, calories burned predictor) and is built using a methodology that ensures communication with stakeholders, implements theories that help identify key characteristics and evaluation metrics, and puts in place behavioral intervention (nudge theory) to ensure the patients stick to their self-management plans in the long term.

## 3. Behavioral Intervention: Nudge Theory

Nudge theory is a collection of behavioral intervention methods that was suggested by [44]. Thaler and Sunstein describe nudge to be “any aspect of the choice architecture that alters people’s behavior in a predictable way without forbidding any options or significantly changing their economic incentives”. “Nudging” is not new as it builds on prior identified theories in the psychological and sociological space dating back more than a century [45].

However, there are two distinct features that are at the root of every valid nudge idea:

1. As pointed out by Thaler and Sunstein, nudges derive knowledge from “behavioral economics and social psychology to explain why people behave in ways that deviate from rationality” [44,45];
2. The political philosophy—libertarian paternalism, where individuals are “actively guided in their best interest but they remain at liberty to behave differently” [45].

When a deeper look is taken at the aforementioned features, it can be seen that nudging is an intervention method that does not “forbid” items but rather aims to subtly suggest otherwise, without the individual actively thinking about it. Thaler and Sunstein provide an example of ways in which one can think of a nudge in relation to dietary choices, where they suggest that “placing fruits at eye level counts as a nudge. But banning junk food is not” [44]. These further highlight that nudges are not a forced mandate that must be followed but rather an appealing alternative suggestion.

From a public health standpoint, the lack of self-management among patients with diabetes has placed a burden on the health systems [4]. Yet patients disregard the health warnings and continue to make poor short-lived dietary decisions without considering the larger picture [46]. There has been research undertaken that looks into nudge theory and how it can affect dietary/self-management behavior [45,46,47]. A systematic review conducted by Arno and Thomas that aimed to “identify the efficacy of nudge theory strategies in influencing adult dietary behavior” concluded that there was on average a 15.3% increase in healthier dietary or nutritional choices made by the participants across 42 studies and that nudge strategies were successful in improving individuals’ dietary choices [46].

With research suggesting that technological self-management intervention is already being refused by patients [48], there is still potential that can be used to leverage the current research conducted in the space of nudge theory to encourage individuals to better manage their diabetes. While there has been research into nudging individuals to make better dietary choices, there is still a clear lack of solutions where nudging is applied to a holistic diabetes self-management solution to better promote among individuals improved self-management of their diabetes rather than a specific outcome. The above being said, a systematic review conducted by Kwan et al. [49] looked at “nudge theories and strategies used to influence adult health behavior and outcome in diabetes management”, and the study highlighted five types of nudging techniques used by the reviewed papers:

1. ***Framing:*** The presentation of a subject matter in order to subtly influence an individual’s choice and behaviors [49];
2. ***Reminders:*** Use of smartly constructed reminders [49];
3. ***Gamification:*** Using the principle of gameplay mechanics to influence behavior [49];
4. ***Social modeling:*** E.g., running group sessions where individuals learn from each other’s experiences [49];
5. ***Social influence:*** Use of external factors as a point of influence, e.g., a weekly summary of the patients’ performance, in relation to a group of individuals [49].

The aforementioned systematic review concluded with the message that nudge theory can cause a positive impact on diabetes intervention, but the authors highlighted that each of the nudging techniques only works in parts [49]. For example, the review suggests that the studies that used reminders as their primary nudging method performed well when it came to medical adherence but proved ineffective in improving the patient’s diet. In contrast, framing was recognized to be the most successful method to influence the patient’s diet but it failed to affect their physical activity habits. This outcome of this systematic review reveals the need for a personalized solution that takes a mixed nudging approach to best address the area of diabetes self-management. Using a personalized nudging approach (different types of nudges based on what requires nudging, e.g., diet = framing, medication adherence = reminders) that can take advantage of various contexts and provide personalized nudging at the appropriate time, coupled with personalized recommendations, can potentially help to improve a patient’s self-management outcome.

## 4. Adapted 4-Cycle Design Science Research Methodology for Design of a Diabetes Self-Management Platform

To ensure the diabetes self-management system proposed is designed and developed in a responsible manner, the solution was co-designed and co-developed using the adapted 4-cycle DSRM (A4C-DSRM) for the diabetes self-management platform [9]. A4C-DSRM is an adaptation of the original 4-cycle design science research methodology (DSRM) [50]. The DSRM revolves around the aim of bundling multiple sociotechnical artifacts, spanning software, process, computer algorithms, and systems, with the goal to improve and/or solve the problem at hand [50]. The A4C-DSRM, while retaining the 4-cycle structure, has been adapted to the diabetes self-management space with Australia’s healthcare system in mind, while also emphasizing on the involvement of clinicians and the patients, which allows for the co-design and development process to occur. By incorporating both a clinical/healthcare professional perspective and a patient context, specifically in the diabetes self-management space, the adapted DSRM allows for an accurate list of patient needs with a clinician backing. Further, due to the co-design cycle found in the A4C-DSRM, this methodology ensures that the implemented features are fit for purpose. The patients will be involved during the evaluation stage of the development and due to the iterative nature of A4C-DSRM, the system will be modified based on patients’ feedback until the requirements are met. 

To ensure the co-designed and developed system is fit for purpose, an adapted task technology fit (ATTF) is followed. The ATTF model was also developed by [9], but it is an adaptation of the original task technology fit model (TTF) [51], which is a well-known theory that is used to guide the fit-for-purpose evaluation of information systems. TTF follows a fit-viability model, which allows researchers to understand and measure the readiness of the organization for technology adoption and the capabilities of the systems to optimally perform the required task [51].

In the ATTF model found in [9], the external factor identifies that the Australian healthcare system is an entity that has the ability to affect all organizational and individual factors. Organization factors identify influences caused by clinics and hospitals, while individual factors are specific to the healthcare professionals and patients that will be in contact with the system in one way or the other. By understanding all the factors that will affect the system at hand using the ATTF model, we are able to measure the “fit” of the system by matching the original identified requirements with the functionalities that the system contains. This will ultimately indicate not only the translation of said requirements but also the performance factors, such as timeliness, reliability, and accuracy.

## 5. Diabetes Self-Management Platform Architecture

**Figure 1:**
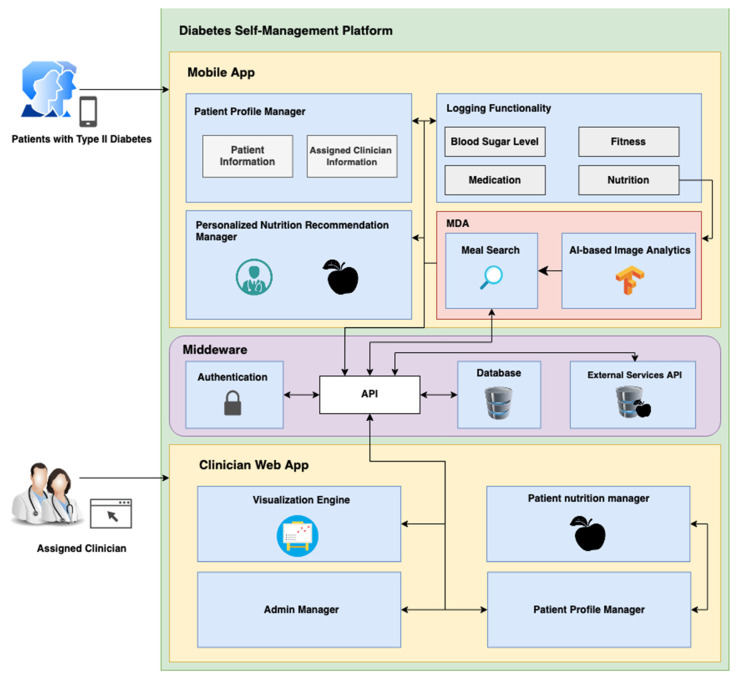
Diabetes self-management platform architecture.

The diabetes self-management platform aims to be a personalized holistic self-management platform that covers the needs of both the patient and the clinician. To identify the requirements and development of this platform, the patients and clinicians were involved to better understand the problem. From a high-level, the platform is divided into two user interactable parts: first, it is a cross-platform *mobile app*, and second, it is a *clinician web app*. They both interact with various *middleware* components. The platform architecture found in Figure 1 highlights the detailed overview of the structure and the processes within the diabetes self-management platform. The platform architecture can be detailed as follows:

### 5.1. Diabetes Self-Management: Mobile App

The diabetes self-management platform’s mobile app contains various components that were identified by the patients and clinicians as a requirement during the co-design cycle of the A4C-DSRM. The following provides greater information on what is described in the diabetes self-management mobile app section of Figure 1. 

*Patient Profile Manager* allows access to crucial patient information, such as name, age, phone number, and any allergies, and it also contains information about the assigned clinician. 

*Logging Functionality* highlights the set of items that the patients need to log in the app. The proposed diabetes self-management app logs the following: blood sugar levels (logged via a Bluetooth glucometer or manual entry), fitness activities participated in (manual entry), medication that has been consumed (manual entry), and any meals or snacks that were consumed (entry through *Meal Search, MDA*, or *Personalized Nutrition Recommendation manager*). 

*Personalized Nutrition Recommendation* contains the clinician’s personalized nutrition recommendation list for the patient. As a part of this, through the clinician web app, the clinician is able to curate a list of meal and snack recommendations personalized for each patient. The patient is able to view this list within the mobile app and is able to log that meal or snack if they have consumed it. 

*Meal Search* allows the user to search the name of a meal or snack item that they would like to add to their log. While doing so, the meal search functionality returns the nutritional composition information of the searched meal before the user is able to log it. 

*Macronutrient Detection Algorithm (MDA)* is the diabetes self-management platform’s ability to handle image-based meal logging by leveraging AI-driven image analytics. Here, we describe how the MDA is implemented inside the diabetes self-management platform’s mobile app. 

This algorithm is spilt into various parts, the first being image classification (*getimage* and *predict*) and second is the nutritional composition of the item (*getnutrition*); see Algorithm 1: MDA Pseudocode. Food classification uses the deep neural network model to perform recognitions, and the output is run through an external API for nutritional composition information.
**Algorithm 1.** MDA Pseudocode1:   **input**: meal image img 2:   **output**: nutrition information of meal image res 3:   **function** getimage 4:     **pass in**: nothing 5:     img ← picked image from gallery 6:     **pass out**: img 7:   endfunction 8:   **function** predict 9:     **pass in**: img 10:      o ← *NULL* 11:      recognitions ← Deep Learning Model (img) 12:      **for each** r in recognitions **do** 13:        **if** o = *NULL* **then** 14:          o ← r 15:        **else** 16:          **if** r.confidence > o.confidence **then** 17:             o ← r 18:          **endif** 19:        **endif** 20:      **endfor** 21:      **pass out**: o 22:   endfunction 23:   **function** getnutrition 24:      **pass in**: name 25:      res ← send request to API for info 26:      **pass out**: res 27:   endfunction 28:   **begin** 29:      img ← getimage 30:      prediction ← predict(img) 31:      **if** prediction ≠ *NULL* 32:        res ← getnutrition(prediction.name) 33:      **endif** 34:      **return** res 35:   **end**

### 5.2. Diabetes Self-Management: Clinician Web App

The diabetes self-management platform’s clinician web app contains various components that were identified by clinicians as a requirement during the co-design cycle of the A4C-DSRM. The following provides greater information on what is described in the diabetes self-management clinician web app section of Figure 1.

*Patient Profile Manager* provides a person the ability to view all of the patient logged information. This includes blood glucose readings, fitness activities, medication, and nutrition intake. 

*Admin Manager* also allows the clinician to view basic patient information, such as name, age, and allergies, but is also able to perform admin duties, such as creating new patient accounts and resetting passwords. 

*Visualization Engine* provides a person the ability to view information as a visualization, general statistics, and uploaded meal images. 

*Manage patient nutrition recommendation* allows the clinician to curate a list of meal and snack recommendation personalized for each patient.

### 5.3. Middleware

The middleware section in Figure 1 identifies all the relevant components that handle requests from the mobile app and the clinician web app. The *API* manages all the *requests* that are created by the diabetes self-management platform. As a part of the middleware setup, all of the requests are *authenticated.* The API is able to make the necessary calls to the database, where all the user-logged information is stored. The *external services API* component is a placeholder to highlight services that are used to retrieve information that is offered by an external entity. An example is the *USDA FoodCentral API* [52], where we are able send requests to get the nutritional composition information of food items.

## 6. Diabetes Self-Management Platform Implementation and Evaluation

This section describes the implementation of the diabetes self-management platform. This includes the diabetes self-management mobile app, the diabetes self-management clinician web app, and also the middleware. We then evaluate the performance of the MDA by using three state-of-the-art deep learning models to assess how well it performs with varying cuisines that were identified during the co-design as a key requirement for supporting personalization of the diabetes self-management platform.

### 6.1. Diabetes Self-Management Platform: Mobile App Implementation

**Figure 2:**
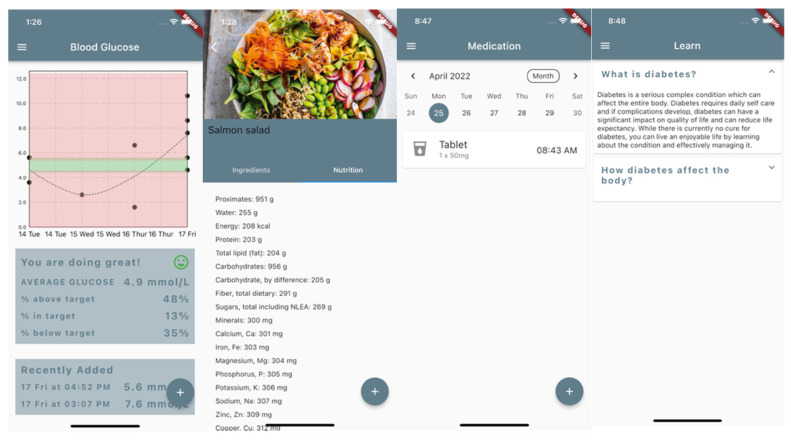
Screenshots of the diabetes self-management app.

The diabetes self-management platform mobile app is the front end with which the patients will interact. The diabetes self-management mobile app is built using the *Flutter SDK* [53], which makes it a *cross-platform* app that can be used in both iOS and Android devices. The patient is required to log in to their personalized user account, which is created by an assigned clinician. The login process is authenticated by the API. The diabetes self-management mobile app contains various features that allow the patient to interact with the app and track their progress in regard to their blood sugar readings, any medication they have been taking, meals they have been consuming, or even any fitness activities in which they have taken part. In Figure 2, some of the described features from the diabetes self-management mobile app are displayed. In addition to the above, the diabetes self-management mobile app implements a *macronutrient detection algorithm* that leverages the use of AI-driven image analytics and other services not only to help the patient better understand the meals they are eating but also to improve the meal logging process by making it simpler.

### 6.2. Macronutrient Detection Algorithm (MDA)—Implementation and Evaluation

As highlighted in the literature, *nutrition management* is a large part of type II diabetes self-management, which has not been addressed by current solutions [10,17]. The diabetes self-management platform offers a few ways for patients to log their nutrition-related information: (a) *Meal Search*; (b) *MDA;* and (c) *Personalized Nutrition Recommendation.* MDA is the diabetes self-management platform’s ability to handle image-based meal logging by leveraging AI-driven image analytics. 

Food classification uses a deep neural network that is a pretrained convolutional neural network (CNN) food classification model named food_v1 [54]. This model has MobileNet v1 as the backbone CNN, and this is trained to identify more than 2000 dishes from images [54]. The model with the pretrained images is then stored on the mobile device to be uploaded for use when necessary.

The model and the list of label map classes are taken and placed within the assets folder of the diabetes self-management mobile app. When the patient goes into nutrition management portion of the app, the model is loaded for use.

- The pretrained model is converted to a mobile-device-compatible TensorFlow Lite (tflite) model and deployed on the mobile app along with the list of classes that the model can classify correctly.
- The model is configured to return three predictions of what it believes the item to be, where the structure of the object includes the label, the confidence, and the class index.
- The result is sorted based on the confidence and provided what the model thinks is the item. To handle incorrect classifications, the *Add Meal* page allows the user to manually edit the item identified.

**Figure 3:**
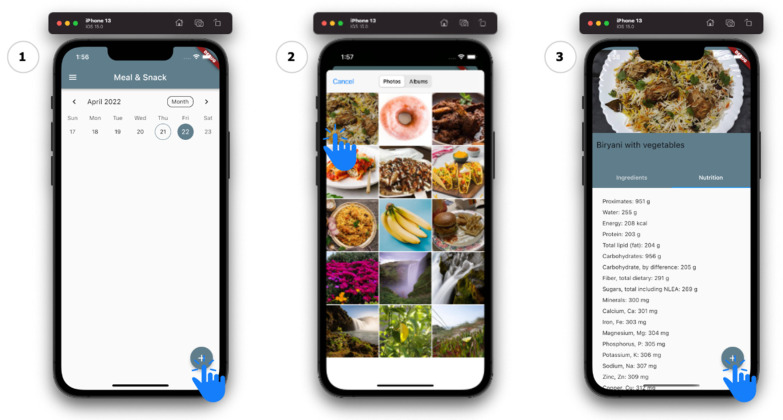
Adding biryani (an Indian meal) through an image with the MDA. (1) Adding an item into the meal & snack page; (2) Select meal image for processing; (3) View meal nutritional composition and add to log if necessary.

To understand the macronutrient information of this item, we take the identified item and pass it through the external API [52]. This allows for the classified item to be translated into an item with nutrition information attached, such as ingredients and the macronutrient information. In Figure 3, we can see a functional use case of how the MDA is used from within the diabetes self-management mobile app. Firstly, from the *Meal & Snack* section of the app, the user is able to press the add icon located in the bottom-right corner; from here the user is able to select the image of a meal that they have consumed or are about to consume; after some background processing, the user is presented with the ingredients and the nutrition composition of the meal; if the user was only looking to better understand the ingredients or the nutritional composition of this item, they are able to simply go back and select another item; however, the user can also continue and add this meal to their log as a meal that they have consumed. This logged meal, along with the selected and analyzed image, is stored in the database by the API, as this will be presented to the clinician through the clinician web app for further analysis and discussions with the patient.

The MDA was identified as a requirement through the co-design process, where it was highlighted that the patients wanted to better understand the meals they were having. Clinicians from the same process felt that this would be a valuable addition to the diabetes self-management app.

This image analytics component also implements features of nudge theory where we nudge the user to make informed choices. Once the photo is processed, the user is met with details regarding the meal they are considering or they have consumed already. The nudge here is that the user may choose a healthier option when they realize that the meals they are viewing are higher in certain macronutrients, such as fats, than they first expected. With this, over time, they will be more knowledgeable about their diet and that may help them subconsciously alter their behavior in how they choose certain meals to consume.

For this evaluation, the dataset found in Table 3 is used. We have outlined various meal images, found online, from three specific cuisines—European, Indian, and Mediterranean. These three specific cuisines were identified by the clinicians during the co-design process as a part of our methodology. These three cuisines were identified as the most common patient diet demographic in their clinics in Melbourne, Australia. By testing the model against specific cuisines, we are also evaluating the performance of the model when put against dishes from various cultures and understanding the bias of using limited or targeted training data, as this highlights the current model support for personalization of meals recommended.

For this technical evaluation, three models were identified as a good fit for this mobile meal classification scenario. The selection criteria for the model were that it needs to be based on a notable architecture and is of a small footprint to be run using the tflite processor:

- Food_v1 [54]
  - Model based off the Mobilenet v1 architecture
  - Trained on 2000+ images, on various dataset
- Monk_v1 Classifier [55]
  - Model based off the Gluon VGG13bn architecture
  - Trained on the Food101 dataset
- Pretrained YOLOv3s [56]
  - Model based off the Efficientnet-B4 classification model
  - Self-collected dataset

The models were measured based on the following performance indicators:

- *Correctness:* A score of 0, 0.5, or 1 was provided based on the correctness of the prediction. This indicator helps better understand the skill of the model in predicting different meals accurately.
  - E.g., If a model predicted “Napolitana Pizza” instead of “Margherita Pizza”, it was given a score of 0.5 as it was able to identify that the item was pizza.
  - If incorrect, the model was given a score of 0.
  - If correct, the model was given a score of 1.
- *Confidence:* In deep learning models, confidence defines the probability of the classification for various classes. In our study, this indicator is used to verify the confidence of the model for a given cuisine.
- *Speed:* Model prediction speed. This indicator highlights the prediction time of a model in a mobile device.

**Figure 4:**
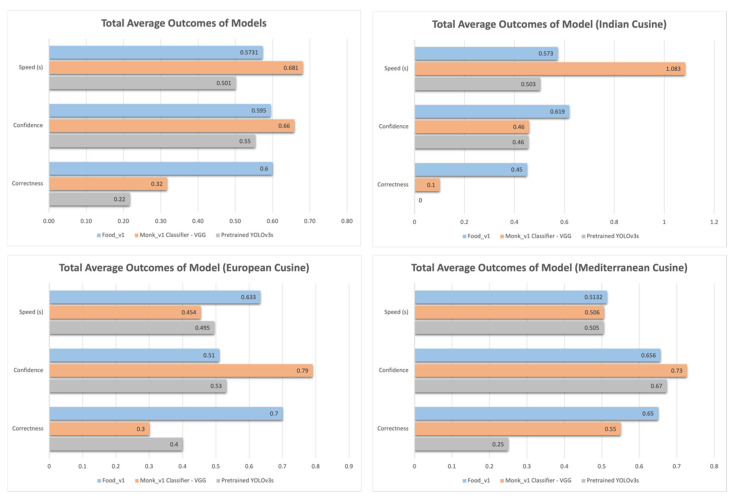
Visualizations of the model comparison outcomes.

For the evaluation, each of the model was loaded into the mobile device one by one. Once loaded, the model was provided with the list of European meals, followed by Indian and Mediterranean meals. By doing so, we were taking pretrained models, with potential biases in their training data, and testing these models against a self-compiled dataset of food items from the clinician-suggested ethnic group diets to see the how well the models perform. This will highlight how well each model performs when given a variety of different food options, which may be different from the items on which the model is originally trained. It is also important to note that the performance indicators also highlight how well these models perform in a mobile device environment, as the models were converted into a tflite format. We are evaluating the models against specific ethnic group diet items as suggested by clinicians and using mobile device centric performance indicators, and the current literature lacks this information. This evaluation is specific to the personalization aspect of the diabetes self-management platform, which requires us to evaluate each of the models with the criteria mentioned above. Each of the results are logged and are visualized in Figure 4.

Through technical evaluation, we are able to see that the Food_v1 model was the most consistent in providing correct classifications. By analyzing the correctness metric, it was identified that the Food_v1 model was able to provide up to 60% accurate classifications across all of the cuisines, while Monk_v1 and Pretrained YOLOv3s were only able to produce 30% and 40% accuracy, respectively. The evaluation also highlighted that the models preform vastly differently depending on the type of dataset that is being use for the evaluation. For Indian cuisine, the Food_v1 model was able to classify 45% of the meals correctly, while Monk_v1 and YOLOv3s were able to classify 10% and 0% respectively. In contrast, the European and Mediterranean cuisines datasets were better classified by all of the models. Food_v1 was able to get 70% of the European dishes and 65% of the Mediterranean dishes correct. Monk_v1 was able to get 30% of the European and 55% of the Mediterranean dishes correct. Pretrained YOLOv3s was able to get 40% of the European dishes correct and 25% of the Mediterranean dishes correct.

Food_v1 is the chosen model for the Phase I implementation of the diabetes self-management platform due to its all-round performance compared to other identified image classification models running on a mobile device.

### 6.3. Diabetes Self-Management Platform: Clinician Web App

**Figure 5:**
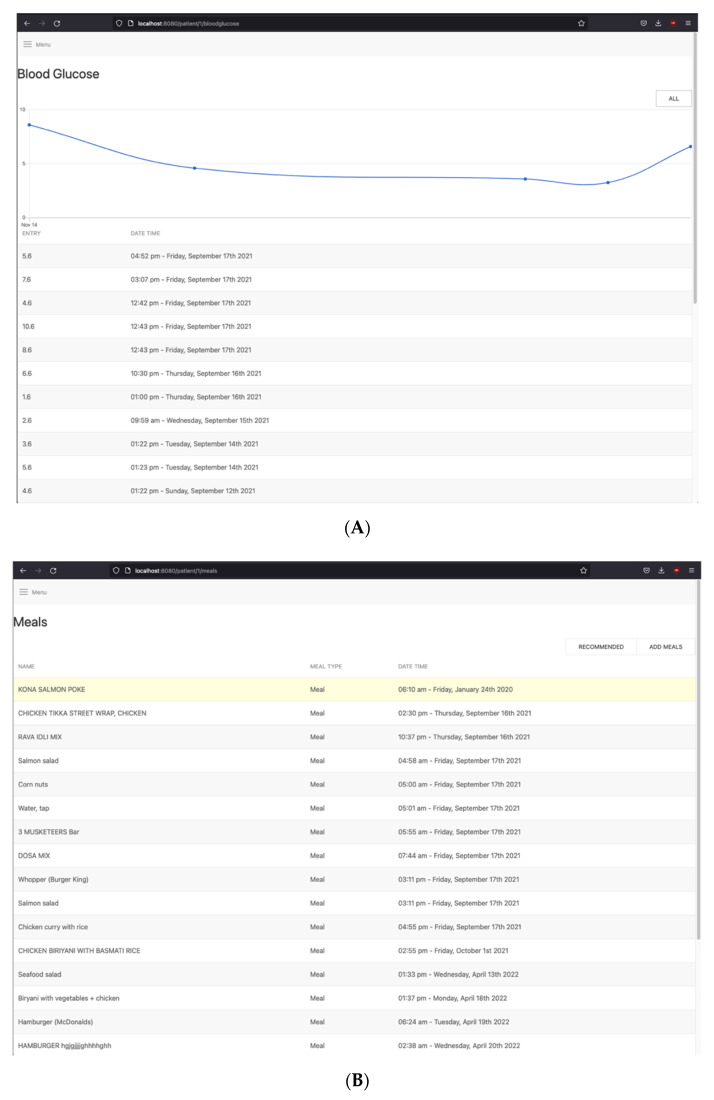
(**A**) Clinician web app—monitor patient blood glucose page; (**B**) Clinician web app—monitor patient nutrition intake page.

The login-based *Clinician Web App* allows for the assigned clinician to view how their patients are progressing with their diabetes self-management. The web app is a single-page application (SPA) created using *Vue.js* [57]. The clinician can view all patient-logged information, which includes blood sugar levels, meals, fitness, and medication. This allows the clinicians to better understand how their patient’s diabetes self-management journey is progressing. The clinician is also able to add or update the personalized nutrition recommendation items through the clinician web app for the patient to view. Figure 5A,B shows sample screenshots of the clinician web app. Figure 5A displays the list of the latest blood-sugar-level readings of a given patient, which is also visualized through a chart. Figure 5B is a list of the latest meal items this patient has consumed.

### 6.4. Middleware

Both the diabetes self-management mobile app and the clinician web app are connected to a backend API. This API is built using NodeJS [58], which was designed and developed to be fast, robust, and secure. The API is responsible for handling the requests from the frontend solutions, making sure everything is secure, and communicating with the database and external APIs. For security, the API has been set up to use Json Web Tokens (JWT) [59] as an authentication middleware, with an Argon2 [60] hashing algorithm to handle storing of sensitive data, such as passwords. The API is set up to only produce authentication tokens with an 8-h expiration for the diabetes self-management mobile app and a 30-day expiration for the clinician web portal. Once the tokens expire, the users will require to log in again to use the platform. Through the API, the USDA FoodCentral API [52] is used to obtain the nutrition composition information.

## 7. Application of the Personalized Diabetes Self-Management Platform in Use

The use-case-based illustrative evaluations presented in this section are developed from Yin’s [61] use-case-based evaluations. This illustrative case study approach allows for the demonstration of the use cases supported by the proposed platform. The use cases described below are developed to illustrate the features of the co-designed diabetes self-management platform.

### 7.1. Use Case 1: Patient–Clinician Pipeline and Nutrition Recommendation 

Jayden is a business executive who was recently diagnosed with type II diabetes. Jayden is of a European background who has no allergies. He was a prediabetic for a while, but during the last GP visit, the clinician confirmed that he in fact needs to take his condition seriously. When working with a dietician, Jayden was instructed to follow a balanced meal plan and partake in regular exercise. He was also asked to check in with his assigned clinician once a month. However, due to Jayden’s work arrangements, he is always traveling for events and meetings, which makes it difficult for him to check in with his clinician and discuss progress. More importantly, he finds it difficult to ensure he maintains a balanced diet on a consistent basis.

By using the diabetes self-management platform, Jayden is able to work with his dietician and come up with meal options that are accessible while he is travelling. The dietician can enter the meal options into the clinician web portal, where recommendations are assigned to Jayden, who is then able to view them on his mobile app when he is out traveling, allowing him to make informed decisions. The assigned clinician is also able to view how Jayden is progressing with his diabetes self-management, through the web portal, which allows for a more accurate insight into Jayden’s progress when the time comes for a formal checkup. 

### 7.2. Use Case 2: Nudge-Inspired MDA

Younis is a brick layer who was diagnosed with type II diabetes three years earlier. Younis is of a Mediterranean background who follows a strict halal diet. Younis has worked with a dietician in understanding what type of meals he should be having and in what portion. However, over the course of his condition, Younis has gained weight and his diabetes has worsened. Younis lacks vital diabetic-related health literacy and has found a lot of the information regarding nutrition management overwhelming and difficult to follow. Due to this, he struggles identifying if the meal he is having is good for him and if the portion is correct.

The diabetes self-management mobile app provides an advanced meal logging functionality where the patient can log their meals from a recommendation list, from a search, or by simply taking a photo of their meal. When a picture of a meal is taken and logged, the diabetes self-management mobile app uses the MDA to identify the meal and what macronutrients this meal contains. The diabetes self-management mobile app is personalized to each of the patients, and as a part of the context, it understands that Younis follows a strict halal diet. Due to this integration, the diabetes self-management mobile app will display a warning if Younis is looking at a meal that has ingredients that are against halal dietary standards. Further, all the images logged are also available for the clinician to view. The clinician will be able to answer any meal-portion-related queries that Younis may have, with examples of meals that he has had. This translates to Younis being able to better understand what a balanced nutrition management plan needs to look like for him and *nudges* him to re-evaluate his diabetes self-management regime. 

## 8. Discussion and Conclusions

This diabetes self-management platform is significantly different to most (if not all) of the current existing solutions that were identified earlier in the literature, as this platform was designed and developed with clinicians’ and patients’ inputs throughout the lifecycle of the research using the A4C-DSRM. By getting both user group inputs, we can design and develop something that is clinically sound, fit for purpose, as well as personalized and patient centered. For the platform containing the patient–clinician pipeline, it is crucial to ensure that the platform continues to be safe to use and patients feel they are able to make progress in their diabetes self-management journey.

In the proposed diabetes self-management platform, there were a few instances where nudges are present. 

*Firstly*, with the implementation of the *Personalized Nutrition Manager* (see Figure 1), the assigned clinician is able to add and curate a list of meal options to the individual. This is always available in the diabetes self-management mobile app for the patient to access at any point in the day. By having a prepopulated list of meals readily available on their mobile device, the patient is more aware of what right kind of meals they should be eating and is inadvertently nudged to do so. 

*Secondly,* through the implementation of the MDA, patients are able to log meals that they are about to consume through an image. However, the MDA can also be used to view the nutritional composition of the meals the patient is about to eat. Hence, whenever the patient logs a new meal item through the MDA, they are aware if the meal they are having is healthy or not. By showing this information, the patient is able to better understand the meal before they eat it and by doing so, they are nudged to eat healthier and make better decisions. 

*Thirdly,* the patient–clinician communication pipeline also acts as a form of nudge. This is because the clinician is able to view all of the patient-logged information through the clinician web app, which includes the meals the patient has eaten. If the patient opts to use the MDA, the clinician will also be able to view the image of the meal the patient is having. This means clinicians will be able to have more honest conversations with their patients about their meals and so the patients are nudged to make improved nutrition choices.

The exemplars from the use-case-based illustrative evaluations further demonstrate some of the reasons behind the need for a personalized diabetes self-management platform. Jayden, from the first use case, highlights some of the common problems that the diabetes self-management platform solves. Jayden is instructed by his GP to take his prediabetic condition more seriously as it is worsening and his dietician recommends that Jayden follow a balanced meal plan along with regular exercise. However, due to his work arrangement, for Jayden to follow a balanced mean plan is difficult. Some problems he may face is that over time his meal plan may become out of date as he has not been able to go in for a meeting with his dietician or he may even lose his diet sheet, which has crucial information about how Jayden can continue to improve his condition. 

Given the popularity of and people’s reliance on smartphones [13], these provide the opportunity where busy individuals, such as Jayden, are always able to access their meal plans compiled by their dieticians. This ensures that, for example, whenever Jayden has his smartphone readily available, he also has with him the nutrition recommendation list from his dietician readily available. This allows Jayden to make informed decision when it comes mealtimes. Current procedures where clinicians have the patients follow static diet sheets and have the patients carry the diet sheets with them while traveling to ensure they make the right decisions leaves room for unfortunate situations and challenges to arise, such as a patient losing the diet sheet or missing an appointment, so the diet sheet is no longer valid. Hence, the diabetes self-management nutrition recommendation system is designed so that it can also be easily updated by the clinician using the accompanied web portal, which means that Jayden can have a teleconsultation appointment with his clinician and the updates made to his meal plan resulting from that appointment are instantly available on his mobile app once the clinician has processed them on the web app. 

The second use case, of Younis, highlights the importance of building a diabetes self-management platform that is accessible for everyone. Younis is a tradesperson who struggles with his diabetes. Over the course of his self-management journey, his diabetes has only worsened. Younis has said that he struggles in identifying if the meal he is having is good for him and finds diabetic-related health literacy difficult to follow. Through Younis’s case study, it is clear that the current structured self-management regime is flawed for his use case. As Younis highlighted, this could be due to him not being able to better understand what his nutrition needs to look like. Through the diabetes self-management platform, Younis will be able to take advantage of the advanced AI image analytics features, which will allow him to take an image of what he is having throughout the day and see the approximate nutritional composition of the said items so Younis can better understand his diet.

The image analytics engine also saves on the platform the images Younis has taken. These images are accessible by his assigned clinician. By having incorporated the image analytics engine, we are allowing for individuals such as Younis to better understand their meals but at the same time, we are having the clinicians better understand what their patients are consuming. The clinician can view the images and improve their nutrition recommendation targeted toward Younis as they better understand the type of meals Younis likes and are able to have conversations with Younis about his diabetes self-management journey and use Younis’s past meal images to reinforce positives and negatives since they last met. This also allows for Younis to better understand what he should be improving on in his diet, as the conversations that the clinicians are starting are backed by Younis’s own past meals. This will help Younis validate and understand which meals he is having are good versus which need attention.

Research has suggested that teleconsultations with clinicians have risen substantially after the outbreak of the COVID-19 pandemic [62]. The diabetes self-management platform is able to provide better support in such crucial situations. Regardless of the situation, if the patient is able to attend a teleconsultation appointment with their assigned clinician, the diabetes self-management platform ensures that the clinician is presented with all the up-to-date information as logged by the patient through their diabetes self-management mobile application, allowing for the clinician’s decision-making to be backed by historical patient data rather than just verbal claims by the patient. This helps improve patient–clinician communications as patient claims are now accompanied by patient data, which ultimately helps the clinician better understand how the patient is progressing in their diabetes self-management journey and work with them to improve it. Use cases presented in Section 7 further provide an insight into how the diabetes self-management platform can be beneficial for the patients and clinicians. However, while the case-study-based illustrative demonstration validates the features of the diabetes self-management platform, we are yet to conduct a pilot study using patients. While this can be seen as a potential limitation of the study, the platform is still in its development stages. We are currently working with a prominent hospital in Melbourne, Australia—to obtain an ethics approval so we can run a pilot study as a part of the next phase of this study.

The model used as a part of the MDA is Google’s AIY, Food_v1 CNN model [54]. This model is able to identify 2000+ dishes as per its training dataset [54]. While the model classifies within its trained dataset quite well, it is observed that the dataset used by Google to create the model is skewed to meals that are available in North America [54], which makes this model effective for diet options available in North America but may not translate to its use by Australia’s multicultural population, with its diverse food options [63]. This was seen during the technical evaluation (see Figure 4), where Food_v1 achieved a 70% accuracy for European meals but only 45% accuracy for Indian meals. Monk_v1 and YOLOv3s also gave similar results, where European dishes were better classified compared to Indian dishes. This highlights the discrepancy when it comes to model training data, where more generic datasets fail to address diversity in meals. By creating a model that is highly performant in predominantly European/Western cuisine, but not in other cuisine, we are creating a rigid and generic model that does not respond to meals from other cultures. The evaluation also captured that the model found it difficult to pick up differences between similar-looking dishes in the absence of a relevant context. For example, an Indian dish called “vada”, which looks similar to a donut/bagel, was identified by the model as such. If the model is able to understand the user context, where the person processing the image has a preference toward Indian diet, we may be able to make better classifications and prioritize different variants of models based on the relevant preferences rather than defaulting to a generic model. The model may also need to be merged with other model architectures and training dataset to achieve improved classifications overall. 

By integrating the user context with the deep learning model, we might be able to provide more personalized results, which brings the potential of building an advance AI model and integrating the patient context defined in the diabetes self-management solution. By doing so, we are giving the model a chance to understand not just the nutrition aspect but also other contexts made available through patient logging, for example, their blood sugar levels and fitness habits. This will allow models as such to provide a greater level of insight into how the patient is progressing with their diabetes self-management journey, provide context-aware nudging as a part of the intervention strategy, and also be able to make improved nutrition recommendations based on the patient’s current context (e.g., calculating effects on blood glucose levels based on a meal’s nutrition composition), allowing for more personalized care. 

Going forward, we will continue to design and develop a *nutrition recommendation system* that will attempt to be more context aware and ultimately provide more personalized care to the patient. We will also be focusing on running a clinical trial on a targeted cohort of patients with type II diabetes from the Indian, Mediterranean, and European backgrounds to help us better understand the impacts of the personalized diabetes self-management platform. Any policy-related implications that will help further the development of the research will be studied.

## Figures and Tables

**Table 1:** Nudge-theory-related studies.

Author(s), Year	Study Design	Sample Size	Duration	Intervention(s)	Outcome
**Charlene et al. 2018** [33]	c-RCT	*n* = 213	2 years	Group 1: Usual care. Group 2: Coaching only. Group 3: Coaching and patient care provider portal (PCPP). Group 4: Coaching, PCPP with decision support.	Reduced HbA1c. Reduced physician visits. Unknown effect on diabetes, distress, depression, blood pressure, and lipid values.
**Saponaro et al. 2021** [34]	Pilot study	*n* = 33	2 weeks	Just-in-time notifications and messages (smartphone + Fitbit). Fitness activity suggestion.	Participants were more active. Nudges worked better when participants were at work versus home.
**Daitaro et al. 2020** [35]	-	-	-	Recommendation Material 1 (RM1): General advice covering easy, attractive, social, and timely (EAST) framework. Recommendation Material 2 (RM2): Personalized patients’ risk against potential risk factors.	RM 2 had higher uptake of the CRC test.
**Elad et al.** **2017** [36]	Study	*n* = 27	26 weeks	Once-a- day and once-a-week messages created by the RL algorithm and pushed to the mobile app.	Overall improved adherence to exercise in diabetic patients. Reduced HbA1c. Increased activity and pace of walking.
**Rabbi et al.** **2015** [37]	Study	*n* = 16	14 weeks	Three weeks of no nudge (baseline). Four weeks of 8 generic prescriptive suggestions from a list of 42. Seven weeks of targeted suggestions. Prioritized low-effort suggestions.	High suggestion adherence. High user satisfaction. Low-effort suggestions had higher actionability rate.

**Table 2:** Diabetes self-management app comparison.

Name	Research		Feature Set																			
	Published	Theory	Medication			Blood Glucose (BG)				Fitness			Nutrition						Clinical			BI
			Log Medication	Add medication by Search	Custom Medication	Log BG Levels	BG Visualization	Set BG Goals/THRESHOLDS	BG Statistics	Log BP Levels	BP Visualization	Calories Burned Estimator	Log nutrition Content	Search Online for Meals	Custom Meals	Nutritional Info of Meal	Nutrition Planner	Nutrition Recommender	Support Network (Coach, etc.)	Assigned Clinician Info	Remote Clinician Monitoring	Any Behavioral INTERVENTION
MySugr	✓ [42]	✕	✓	✕	✓	✓	✓	✓	✓	✕	✕	✕	✓	✕	✓	✕	✕	✕	✕	✕	✕	✕
Blood Sugar Log	✕	✕	✓	✕	✓	✓	✓	✕	✓	✕	✕	✕	✕	✕	✕	✕	✕	✕	✕	✕	✕	✕
Glucose Tracker & Diabetic Diary	✕	✕	✓	✕	✓	✓	✓	✓	✓	–	✕	✕	–	✕	✕	✕	✕	✕	✕	✕	✕	✕
Diabetes:M	✕	✕	✓	–	✓	✓	✓	✓	✓	✕	✕	✕	✓	✓	✓	✕	✕	✕	✓	✓	✓	✕
Glucose Buddy Diabetes Tracker	✕	✕	✓	✕	✓	✓	✓	✕	✓	✓	✓	–	✓	–	✓	✓	✕	✕	✓	✕	✕	✕
One drop diabetes management	✓ [43]	✕	✓	–	✓	✓	✓	✓	✓	–	–	–	✓	✓	✓	✓	–	✕	✓	✕	✕	✕
Blood sugar monitor by Dario	✕	✕	✕	✕	✕	✓	✓	✓	✓	–	✕	✕	✓	✓	✓	✓	✕	✕	✕	✕	✕	✕
Blood Glucose Tracker	✕	✕	✓	✕	✓	✓	✓	✓	✓	✕	✕	✕	✓	✕	✓	✕	✕	✕	✕	✕	✕	✕
ForDiabetes: diabetes self-management app	✕	✕	✓	✕	✓	✓	✓	✓	✓	–	–	–	–	✕	✕	✕	✕	✕	✕	✕	✕	✕
Glucose—blood sugar tracker (iOS only)	✕	✕	–	✕	–	✓	✓	✓	✓	✕	✕	✕	–	✕	✕	✕	✕	✕	✕	✕	✕	✕

**Table 3:** Meals from various cuisines used for model evaluation.

Meals		
European	Indian	Mediterranean
Burger	Biryani	Dolmas
Chicken Burger	Butter Chicken	Falafel
Donut	Daal	Greek Salad
Grilled Cheese	Dosa	Paella
Chicken Parmigiana	Idli	Pasta Fettuccini
Cheese Steak	Naan	Pasta Napoli
Roast Chicken	Paani Puri	Pita
Sandwich	Papadum	Margherita Pizza
Sausages	Mutton Curry	Ratatouille
Steak	Vada	Risotto

## Data Availability

Not applicable.
